# Case report: Laparoscopic treatment of gastro-omental hemangioma with hemorrhage: two cases reports and review of literature

**DOI:** 10.3389/fsurg.2023.1156337

**Published:** 2023-05-05

**Authors:** Long-Kuan Yin, Jian-Jun Liu, Yi-Guo Feng, Hua-Yan Yuan, Wei Wang, Xiang-Yu Bai, Pan Wang

**Affiliations:** Department of Gastrointestinal Surgery, Affiliated Hospital of North Sichuan Medical College, Nanchong, China

**Keywords:** laparoscope, omentum, hemangioma, hemorrhage, abdominal apoplexy

## Abstract

**Background:**

Spontaneous hemorrhage of gastro-omental hemangioma is a rare disease. The treatment strategy for this disease changes as it develops. In the acute stage, surgery is the first priority, among which laparoscopic treatment should be the most frequently considered option in large general hospitals. Due to the rarity of this disease, such cases have rarely been reported.

**Case description:**

We present the first report of two eldely cases with gastro-omental hemangioma with hemorrhage by laparoscopic treatment. Both cases were initially admitted with upper abdominal pain, and abdominal computed tomography (CT) scan revealed masses alongside the greater curvature of the stomach. Laparoscopic surgery was conducted immediately in both patients. The two cases recovered well after surgery, and no obvious abnormalities were observed in the follow-up period.

**Conclusion:**

Gastro-omental hemangioma rupture remains an uncommon cause of intraperitoneal hemorrhage. Timely diagnosis and surgery are paramount for treatment. Medical institutions with the correct technology and equipment should perform laparoscopic treatment to minimize surgical trauma and promote rapid recovery of patients with abdominal apoplexy.

## Introduction

Spontaneous hemorrhage of gastro-omental hemangioma is not common, and it may occur without any recent history of abdominal trauma, surgery, anticoagulant therapy, or systemic vascular disease. Clinical diagnosis should be made with abdominal B-ultrasound, abdominal CT, abdominal puncture, and other auxiliary examinations. The treatment methods generally depend on the disease development and the patient's conditions. In the onset or chronic phase, clinical observation and medical treatment are routine options, while in the advanced or acute phase, surgical treatment is preferred (1, 2). Recently, several case reports of gastro-omental hemangioma combined with hemorrhage have been reported; however, few studies have reported on laparoscopic surgery for this condition (3, 4). In this article, we present two elderly cases with spontaneous gastro-omental hemangioma with hemorrhage and huge intra-abdominal hematoma treated with laparoscopic surgery, achieving a good treatment effect. Both cases recovered well after operation, and experienced no discomfort in the follow-up period.

## Case presentation

### Case 1

A 61-year-old female was admitted to our hospital on September 16, 2018 due to persistent dull pain in the middle and upper abdomen for > 2 months, with aggravation for 1 day. The pain was relieved after taking an oral anodyne (specific name and dosage unknown) prescribed by a local physician, and no further examination or treatment was given. She denied any history of hypertension, diabetes, hepatitis, tuberculosis, heart disease, abdominal trauma, or operation. Physical examination showed the patient had acute facial features and pallid eyelid conjunctiva, and her skin was clammy and less elastic, with a body temperature (T) of 36.5°C, pulse frequency (P) of 80 beats/min, respiration frequency (R) of 20 beats/min and blood pressure (BP) of 126/80 mmHg. Abdominal moving dullness was positive, and non-coagulated blood was extracted by diagnostic abdomen puncture.

Routine blood examination revealed the following: white blood cell count (WBC) of 9,800/mm^3^ (normal range 4,000–10,000/mm^3^), red blood cell count (RBC) of 3,100/mm^3^ (normal range 3,800–5,100/mm^3^), platelet (PLT) of 130/mm^3^ (normal range 100–300/mm^3^), Hematocrit (HCT) of 0.283 (normal range 0.350–0.450), and a hemoglobin (HGB) concentration of 91 g/dl (normal range 115–150 g/dl). No abnormality was found on coagulation function examination. The above results indicated that the patient had an intraperitoneal hemorrhage combined with moderate anemia.

**Figure 1:**
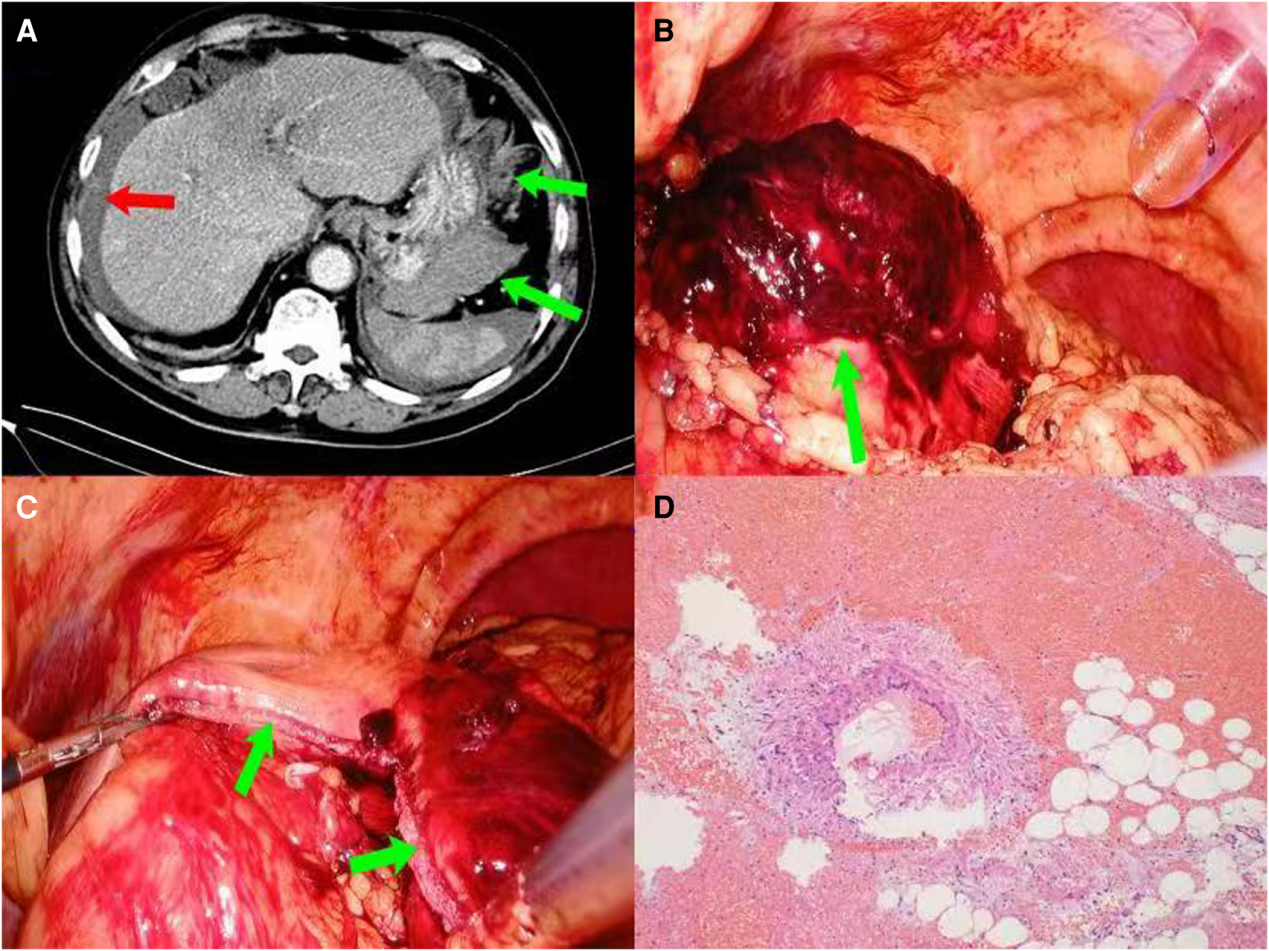
(Case 1) (**A**): enhanced CT of the upper abdomen showing a mass in the greater curvature of the stomach (green arrows), with fluid/blood accumulation around the liver and spleen (red arrow); (**B**): the boundary between the hematoma and the greater curvature of the gastric wall was not clear (green arrow); (**C**): intraoperative resection of the hematoma and part of the wedge-shaped gastric wall (green arrows); (**D**): postoperative pathological examination identified identified an omental capillary hemangioma with hemorrhage (HE × 100).

Abdominal enhanced computed tomography (CT) revealed an effusion in the abdomen and pelvis, suspicious blood accumulation around the liver and spleen, and a slightly patchy high-density shadow on the side of the great curvature of the gastric body, about 9.8 × 6.9 × 6.5 cm in size (Figure 1A). Emergency laparoscopic exploration was subsequently performed. During the procedure, about 1,400 ml of bloody fluid was found in the abdominal pelvic cavity, and an elliptical mass about 10.1 × 8.2 × 8.0 cm in size, with a surrounding blood clot was detected in the omentum of the greater curvature of the gastric body (Figure 1B). The boundary of the mass was unclear, and accompanied by active hemorrhage, which was difficult to separate from the greater curvature of the gastric body. Therefore, the mass and the attached wedge-shaped stomach wall were simultaneously removed by an endoscopic linear cutting stapler (EC60A, Ethicon) (Figure 1C). Hemostasis, anti-infection, and symptomatic treatment were provided postoperatively. Postoperative pathological examination revealed an omental capillary hemangioma with hemorrhage (Figure 1D), and the patient was discharged on postoperative Day 7. No complaints of special discomfort were mentioned, and no abnormal findings were found on routine blood and abdominal CT examination in the 1 year followed-up period (data not shown).

### Case 2

An 81-year-old male was admitted on December 13, 2020 due to upper abdominal pain for 20 h. His medical history revealed hypertension for approximately 10 years, with a maximum blood pressure of 180/110 mmHg. He was receiving Nifedipine to control blood pressure. The patient had no history of hepatitis, tuberculosis, heart disease, diabetes, trauma, surgery, or blood transfusion.

Physical examination indicated acute facial features, clammy and weakened elastic skin, with T 36.7°C, P 90 beats/min, R 25 beats/min and BP 138/90 mmHg. His abdomen was flat, and the middle and upper abdomen had a moderate tenderness and rebound pain, without muscle tension; however, abdominal moving dullness was strongly positive, and non-coagulated blood was obtained by diagnostic abdominal puncture. Routine blood examination showed the following: WBC 9,800/mm^3^, RBC 3,200/mm^3^ (normal range 4,300–5,800/mm^3^), PLT 91/mm^3^, HCT 0.311 (normal range 0.400–0.500), HGB: 107 g/L (normal range 130–175 g/dl), and a normal coagulation index. The above results confirmed that the patient had an intraperitoneal hemorrhage combined with moderate anemia.

**Figure 2:**
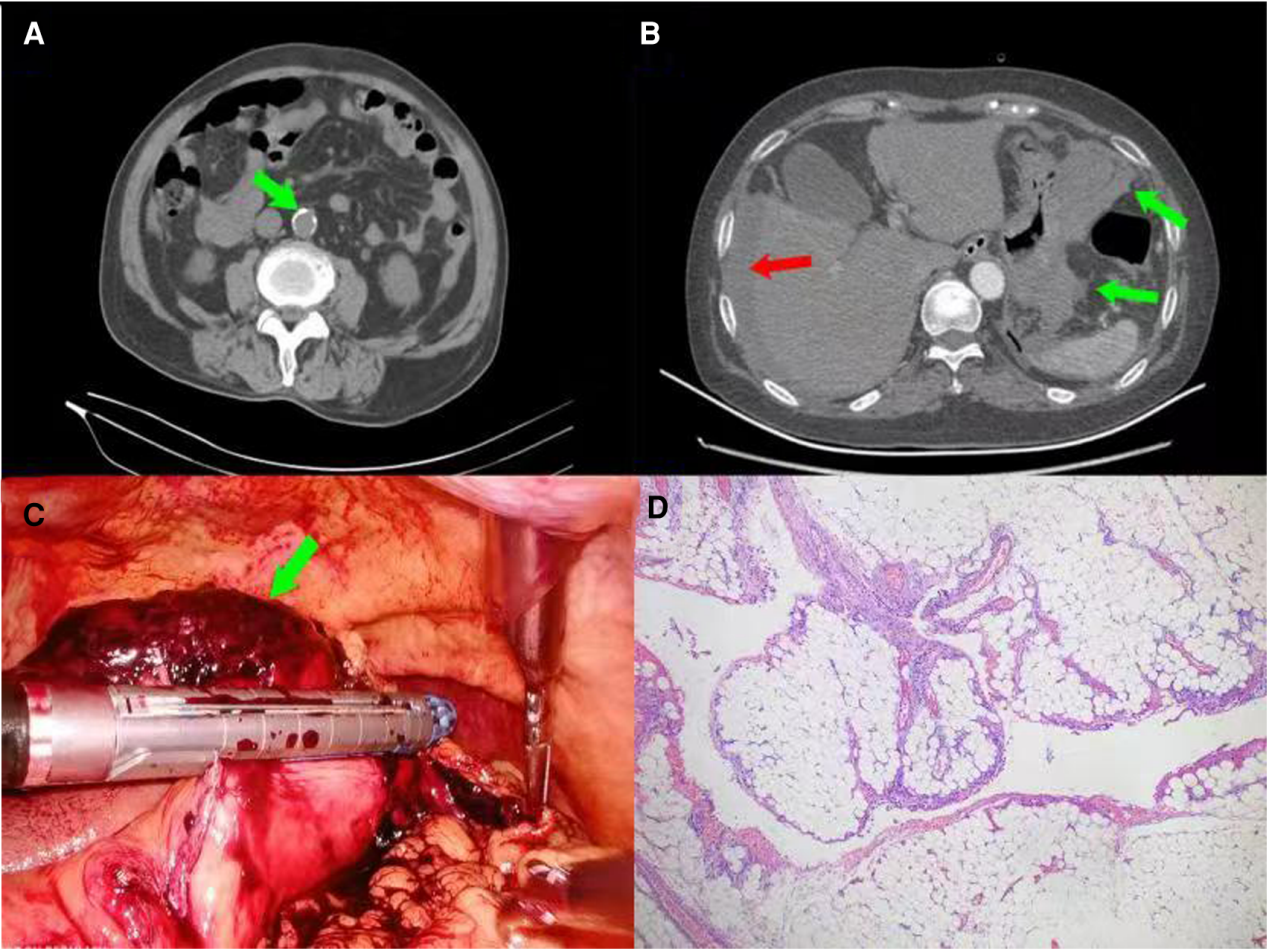
(Case 2) (**A**): CT-scan showing the formation of calcified plaques in the abdominal aorta (green arrow), bilateral common iliac artery, bilateral internal iliac artery, and right external iliac artery, partially accompanied by mural thrombosis; (**B**): enhanced CT of the upper abdomen showing hematoma formation in the upper left abdominal cavity, an unclear boundary between the hematoma and stomach (green arrows), and abdominal pelvic fluid/blood (red arrow). (**C**): The procedure was conducted by removing the mass and partly attached gastric wall with an endoscopic linear cutting stapler (EC60A, Ethicon). (**D**): Postoperative pathological examination showing a ruptured cavernous hemangioma with hemorrhage and thrombosis (HE × 100).

Abdominal enhanced CT scan revealed the following: 1. Obvious local lumen stenosis was observed at the beginning of the Celiac axis, with a few calcifications on the wall. This was considered as atherosclerosis. Calcified plaques had formed in the abdominal aorta, bilateral common iliac artery, bilateral internal iliac artery, and right external iliac artery, partially accompanied by mural thrombosis (Figure 2A). 2. Hematoma had formed in the upper left abdominal cavity. The boundary between the hematoma and the greater curvature of the gastric wall was unclear, and abdominal pelvic fluid/blood was detected (Figure 2B). The hematoma was 10.2 × 9.5 × 3.3 cm in size, and located in the greater curvature of the stomach. Their boundary was not clear. Surgery involved removal of the mass and partial attachment of the gastric wall with an endoscopic linear cutting stapler (EC60A, Ethicon) (Figure 2C). The postoperative pathological results confirmed the diagnosis of ruptured omental cavernous hemangioma accompanied by thrombosis (Figure 2D). The patient was discharged on postoperative Day 8. No obvious abnormalities were reported and no abnormal findings were detected by blood routine and abdominal CT examination in the 6-month outpatient follow-up period (data not shown).

## Discussion

Intraperitoneal bleeding can have many causes, including trauma, iatrogenic or ruptured aneurysm, gynecological disease, malignant tumors, and inflammatory or autoimmune processes, but spontaneous or idiopathic bleeding is rare. Abdominal apoplexy is a type of acute abdomen usually caused by unknown factors, and is accompanied by intra-abdominal vascular spontaneous rupture and retroperitoneal hemorrhage. The pathogenesis of abdominal apoplexy is still unclear; however, a few reports have reported possible causes as follows: 1. Abdominal organ vascular malformations, visceral tumors, abnormal coagulation function, liver cirrhosis, hypertension, arteriosclerosis, rheumatism, immune system diseases, and so on. It has also been documented that visceral vascular rupture can lead to abdominal bleeding. Kumari J et al. (5) reported the occurrence of a case of spontaneous intraperitoneal bleeding without obvious inducement during pregnancy. Pancreatic tumor fibroma has also been reported as a rare cause of abdominal bleeding (6). In addition, rupture of large liver tumors and female uterine fibroids may also lead to accidental bleeding in the abdominal cavity (7, 8). Another study reported that some abdominal varicose veins can lead to spontaneous abdominal bleeding (9); 2. A small number of young people with no definite cause may also be at risk of abdominal apoplexy (1); 3. The rupture of Gastro-omental hemangioma is another infrequent cause of intraperitoneal hemorrhage (10), also as reported in this paper.

Previous reports have indicated that patients with abdominal apoplexy should undergo auxiliary examination, including blood routine examination, abdominal B-ultrasound, abdominal x-ray, abdominal CT, computed tomography angiography (CTA), angiography, intervention, etc (3–7). Surgeons may choose different treatment measures for abdominal apoplexy, as follows: A) Conservative treatment: thrombin, blood transfusion and rehydration are selectively administered to patients with an unclear diagnosis, small amount of bleeding, or stable vital signs (11); B) Surgical treatment: Exploratory laparotomy is strongly recommended for those with clear surgical indications who suffered from a large amount of blood loss, and a poor or ineffective response to conservative treatment. Surgical treatment can be roughly divided into two types: laparoscopic exploration and open laparotomy (12); C) Interventional therapy: Patients with definite bleeding sites with stable hemodynamics could underwent if the bleeding site is special or with high risk of surgical methods (6).

In this article, we reported two cases of gastro-omental angioma with spontaneous rupture and bleeding. As shown in Table 1, due to the rarity of this factor as a causes of abdominal apoplexy, there are currently 8 cases reported in the literature (3, 4, 10, 13–17). The specific pathogenesis of this disease is still unclear, but the following high-risk factors need to be considered when coming to the diagnosis: 1. Elderly patients with cardiovascular disease, such as hypertension, arteriosclerosis, hyperlipidemia, coronary heart disease, etc, who take long-term oral anticoagulant medicine should be considered for the possibility of gastro-omental angioma rupture (18). For example, the second case in this paper suffered from primary hypertension, with a blood pressure occasionally reaching up to 180/110 mmHg, and a past history of cerebral infarction and subsequent long-term oral aspirin anticoagulation therapy; 2. This disease can also occur in some patients without high-risk factors and no obvious triggers (19); 3. Patients with congenital vascular malformations can also have abdominal apoplexy, especially in the case of overeating, alcoholism, etc. Several studies have reported a mortality of up to 100% in patients with abdominal apoplexy treated with conservative methods (18, 19). If the bleeding site can be found by exploratory laparotomy, and the bleeding can be controlled effectively, the mortality rate can be reduced to around 8.6%; however, it can reach up to 54.8% if the bleeding site cannot be detected. The main causes of death are hemorrhagic shock and its corresponding complications (20).

**Table 1:** The summary of previous reported cases of omental hemangioma.

Author	Year	Age	Sex	Location	Interventions	Intraperitoneal blood loss	Pathological examination
Ritossa (10)	1989	11Y	F	Greater omentum	Open surgery	N/A	Cavernous hemangioma
Shih (13)	1995	10Y	F	Greater omentum	Open surgery	N/A	Malignant hemangioendothelioma
Ratan (14)	1999	14Y	F	Greater omentum	Open surgery	None	Epithelioid hemangioendothelioma
Chateil (3)	2000	5M	F	Greater omentum	Laparoscopic surgery	None	Capillary haemangioma
Chung (15)	2000	25Y	M	Greater omentum	Open surgery	None	Hemangiopericytoma
McHugh (16)	2002	14Y	M	Greater omentum	N/A	None	N/A
Vyčítal (17)	2019	43Y	M	Greater omentum	Open surgery	None	Hemangiomatosis
Nagano (4)	2020	63Y	M	Lesser omentum	Laparoscopic surgery	None	Capillary hemangioma

Y, years old; M, momth; F, Female; M, Male; N/A, not available.

In this article, although the vital signs and hemodynamics were relatively stable in both cases, we chose to perform operation as both patients were advanced in age, and their tolerance to bleeding was poor. Moreover, abdominal B-ultrasound and CT examination both confirmed heavy intra-abdominal bleeding, and the bleeding sites were undefined. In fact, if the patient's conditions are permitted, further preoperative examinations, including CTA, MRI or upper GI Endoscopy, ect, should be conducted to identify the source of bleeding for these types of cases. In the follow-up (at 6 and 12 months in cases 1 and 2, respectively) no obvious abnormalities were reported, indicating a good prognostic outcome.

Taken together, this is the first report of two eldely cases with gastro-omental hemangioma hemorrhage treated with laparoscopy. Clinicians should consider the possibility of spontaneous rupture of gastro-omental hemangioma when diagnosing patients with high-risk factors for abdominal apoplexy. Furthermore, laparoscopic exploration should be conducted in medical institutions with the relevant experience, as this can greatly reduce the surgical trauma to the patients, while aiding in a quick recovery. Although laparoscopic treatment of abdominal apoplexy achieved good therapeutic effect in the two cases reported herein, in patients where laparoscopic surgery is difficult, the procedure could be converted to open laparotomy or exploratory laparotomy.

## Data Availability

The original contributions presented in the study are included in the article, further inquiries can be directed to the corresponding author.
